# Society Meetings

**Published:** 1915-10

**Authors:** 


### SOCIETY MEETINGS

**Brief reports and announcements of meetings of societies of Western New York, and those of general scope, are requested from Secretaries. Copy should be on hand the fifteenth of the month. Full transactions will be published at cost of composition.**

---

**DOCTOR: Is your Society properly represented here? If not, please bring our standing announcement to the attention of the members.**

---

**The American Medical Editors' Assn.** will meet in N. Y. October 18 and 19, with headquarters at the Hotel McAlpin, under the presidency of H. Edwin Lewis. Joseph McDonald, Jr., is secretary and treasurer, with offices at 92 William Street, N. Y.

---

**The American Laryngological Assn.** held its 27th annual congress at Niagara Falls, Ont., June 1-3, as previously announced. We have received from the Abstract Editor, Emil Mayer, of N. Y., proof sheets of proceedings for publication but, owing to the scope of this Journal and the fact that the matter would fill at least one whole issue, we regret our inability to do so. Any one interested, however, may consult this report at our office or may borrow it.

**The National Assn. for the Study and Prevention of Tuberculosis** has inaugurated a campaign to have every one examined by a physician on December 8. Special instruction in hygiene for school children and the discussion of tuberculosis from over 125,000 pulpits are included in the plan. The Buffalo Express raises the question whether physicians will charge for examinations and whether the tuberculosis propaganda have not been overdone.

---

**The American Public Health Assn., The Sanitary Officers of the State of N. Y. and the N. Y. State Sanitary Officers' Assn.** held a joint congress in Rochester, September 6-10, as guests of the **Rochester Public Health Assn.** 110 addresses and 250 medical and sanitary papers were read.

---

**The American Association of Obstetricians and Gynaecologists,** of which our esteemed predecessor, Lt. Col. W. W. Potter, was long Secretary, held its 28th annual meeting in Pittsburgh in September. Dr. Hugo J. Panzler was elected President and Dr. Herman E. Hayd of Buffalo, Treasurer. Indianapolis was selected for the 1916 meeting.

---

**The Eighth District Branch of the Medical Society of the State of New York** held its tenth annual meeting at Olean, September 21 and 22. The officers were: President, Carl G. Leo-Wolf; Vice-president, Albert T. Lytle; Secretary, Edward A. Sharp; Treasurer, Charles A. Wall, all of Buffalo. Invited guests were: James C. Bloodgood of Baltimore and L. Duncan Bulkley of N. Y. Officers were elected as follows: President, A. T. Lytle; Vice-Presidents, Edward Torrey, Olean; W. Ross Thompson, Warsaw; Secretary, L. C. Lewis, Belmont; Treasurer, F. H. Vanarsdale, Belmont.

---

**The Medical Brotherhood (Fraternitas Medicorum),** has been organized, at the initiative of Dr. S. J. Meltzer of New York, an active member and ex-president of the American Gastro Enterologic Assn., to further the peace movement and international good will and co-operation among medical men, in spite of war. Nearly 1500 physicians have already been enrolled in this country. There are no dues but contributions will be accepted to defray incidental expenses. Physicians are urged to co-operate in this movement. The officers are as follows:

Executive Committee—(Residents of the City of New York) President, Dr. S. J. Meltzer, Member of Rockefeller Institute; Vice-Presidents, Dr. Rufus Cole, Director, Rockefeller Hospital; Dr. S. Josephine Baker, Director, Department of Child Hygiene; First Secretary, Dr. Wm. J. Gies, Professor of Biological Chemistry, Columbia University; Second Secretary, Dr.

Harlow Brooks, Professor of Clinical Medicine, N. Y. University and Bellevue Hospital Medical College; Treasurer, Dr. Robert T. Morris, Professor of Surgery, Post-Graduate Medical School.

---

**The Medical Society of the County of Chemung** held its regular meeting at Elmira, September 21. Scientific Program: Title not announced, H. H. Crum, Ithaca; Our Whooping Cough, Anna Stuart; Heterodox Therapeutics, G. B. R. Merrill; Newer Therapeutics, Wm. Brady. C. F. Abbott, President, C. L. Carey, Secretary.

---

Dr. William Cain entertained the members of the **Elmira Clinical Society** at its Annual Outing, on September 1st. An automobile trip was taken to Breesport, where various games and a corn-roast were enjoyed.

---

**The Western N. Y. Homoeopathic Medical Assn.** met at the J. N. Adam Memorial Hospital September 22, under the presidency of Dr. R. Montford Schley of Buffalo. A demonstration of cases was given by Dr. John H. Pryor of Buffalo. About 50 were present.

